# Evolutionary analysis of *gyrA* gene from *Neisseria meningitidis* bacterial strains of clonal complex 4821 collected in China between 1978 and 2016

**DOI:** 10.1186/s12866-020-01751-5

**Published:** 2020-03-30

**Authors:** Pan Zhao, Li Xu, Aiyu Zhang, Bingqing Zhu, Zhujun Shao

**Affiliations:** State Key Laboratory for Infectious Disease Prevention and Control, National Institute for Communicable Disease Control and Prevention, Chinese Center for Disease Control and Prevention, 155 Changbai Road, Changping, Beijing, China

**Keywords:** DNA gyrase subunit A, Quinolone Resistance-Determining Region, *Neisseria meningitidis* clonal complex 4821, Recombination

## Abstract

**Background:**

*Neisseria meningitidis* (*N.meningitidis*) bacteria belonging to clonal complex 4821 (CC4821) have been mainly reported in China and have been characterized by a high resistance rate to ciprofloxacin (CIP). The aim of this study was to assess the evolution of the DNA gyrase A (*gyrA*) gene from *N.meningitidis* CC4821 strains collected in China between 1978 and 2016. The complete sequence of *gyrA* gene from 77 strains are reported in this study and analyzed in the context of publicly available sequences from *N. meningitidis* of other CCs as well as other *Neisseria* species.

**Results:**

The phylogenetic analysis of CC4821 *gyrA* gene reveals at least 5 distinct genetic clusters. These clusters are not CC4821-specific showing that *gyrA* evolution is independent of CC4821 evolution. Some clusters contain sequences from other *Neisseria* species. Recombination within *N.meningitidis* strains and between *Neisseria* species was identified in SimPlot analysis. Finally, amino acid substitutions within GyrA protein were analyzed. Only one position, 91 (83 in *E.coli gyrA* gene), was linked to CIP resistance. Thirty-one additional putative resistance markers were identified, as amino acid substitutions were only found in resistant strains.

**Conclusions:**

The evolution of *gyrA* gene of CC4821 *N.meningitidis* strains is not dependent on CC4821 evolution or on CIP resistance phenotype. Only amino acid 91 is linked to CIP resistance phenotype. Finally, recombination inter- and intra-species is likely to result in the acquisition of various resistance markers, 31 of them being putatively mapped in the present study. Analyzing the evolution of *gyrA* gene within CC4821 strains is critical to monitor the CIP resistance phenotype and the acquisition of new resistance markers. Such studies are necessary for the control of the meningococcal disease and the development of new drugs targeting DNA gyrase.

## Background

Meningitis is an inflammation of the protective membranes covering the brain and the spinal cord (https://www.cdc.gov/meningococcal). This disease can have multiple causes like bacteria or virus but also fungus, parasite or even non-infectious agent like lupus. Bacterial meningitis can be caused by several types of bacteria including *Streptococcus pneumonia* or *Neisseria meningitidis* (*N.meningitidis*). In addition to *N.meningitidis*, the genus *Neisseria* contains at least 30 distinct species colonizing humans, other mammalians and even insects [1]. *N.meningitidis* are classified through several different schemes, based on serological test (serogroup) or genetic tests (sequence type) [2, 3]. Among the 12 described serogroups, which are based on the structure of the capsule polysaccharide (*cps*), 6 serogroups (A, B, C, X, Y and W) caused the majority of invasive meningococcal disease (IMD) globally [2]. In addition, the strains can be grouped into different sequence types (STs) based on the multilocus sequence typing (MLST) method on 7 genes (*abcZ*, *adk*, *aroE*, *fumC*, *gdh*, *pdhC*, *pgm*) [3]. An ST is characterized by a different sequence nucleotide for at least one of the 7 reference genes. So far, 14,556 STs have been described. Furthermore, the strains that are sharing the sequence of 4 or more genes (identified as a number for convenience) among the 7 genes used to determine STs could be classified into the same clonal complex (CC) [3]. So far, 48 CCs have been described (https://pubmlst.org/neisseria/). The strains that could not be classified into an existing CC were called unassigned (UA).

In China, the strains of *N. meningitidis* of serogroup A with either CC1 or CC5 were responsible for the majority of IMD cases until 2003 [4]. In 2003, an outbreak of a new serogroup C meningococcal disease caused by CC4821 was reported in Anhui province of China. This new hypervirulent clonal lineage did not belong to any of the previously reported sequence types [5]. Subsequently, CC4821 serogroup C became one of the leading lineages across China [4]. Later on, CC4821 became also a dominant lineage among serogroup B strains since the first report in 2005. However, in contrast to serogroup C strains, serogroup B strains have been usually associated with sporadic infections [4]. Analyses of historic isolates showed that CC4821 strains of serogroup B and C strains were isolated as early as in 1978 and were mostly associated with asymptomatic carriers [6].

Two main strategies have been developed to control the meningococcal disease. Vaccines specific to multiple serogroups have been generated and are globally used [7]. Furthermore, as for other bacterial infections, antibiotics have been also used to control the infection. The most common, quinolone and its derivatives like ciprofloxacin (CIP), is targeting the DNA gyrase A (GyrA) which is essential for DNA replication [8, 9]. Quinolone interaction with GyrA has been well studied thanks to the 3D structure of the *E.coli* protein [10]. The mutation of critical sites lead to resistance; these sites are located in the so-called Quinolone Resistant-Determining Region (QRDR) [11].

The aim of this study was to assess the evolution of the *gyrA* gene from CC4821 strains collected in mainland China between 1978 and 2016. The sequences were compared to *gyrA* sequences from *N,meningitidis* with different CCs as well as sequences from other species of Neisseria. Monitoring the evolution of *gyrA* gene, especially the sites involved in antibiotic resistance is capital to control meningococcal disease.

## Results

### Evolutionary analysis of 77 *gyrA* nucleotide sequences from CC4821 *N.meningitidis* strains

**Fig. 1:**
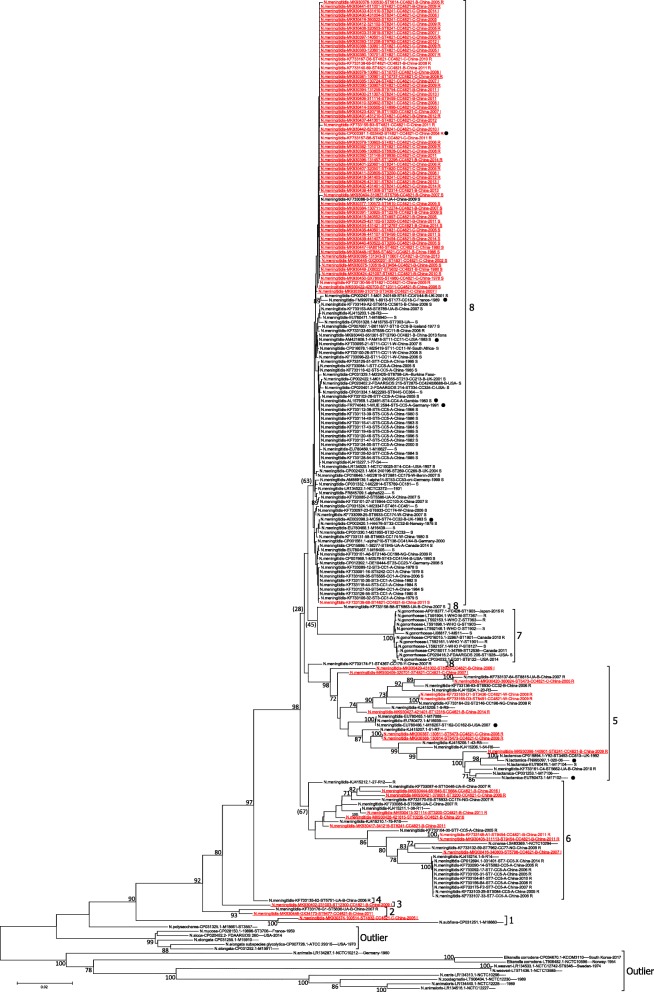
Neighbor joining phylogenetic tree of 226 *gyrA* gene sequences from *Neisseria* strains. Strain name is indicated as follows: species name-GB ID-strain ID-ST-CC-Serogroup-country of collection-year of collection-CIP resistance phenotype. Missing information is indicated by an empty space. For example, *N.meningitidis*-AM889136.1-alpha14-ST53-CC53-*cnl*-Germany-1999 S; *Eikenella corrodens*-CP034670.1-KCOM3110----South Korea-2017. The sequence names from CC4821 *N.meningitidis* strains are indicated in red font. The 77 sequences generated in this study are underlined. The sequences of 9 reference strains are indicated by a black dot. Bootstrap values > 70% are indicated. Bootstrap values < 70% are indicated in parenthesis when necessary. The 9 genetic groups identified in this study are indicated as bracketed vertical lines. CIP resistance phenotype is indicated with R for resistance, S for sensitive and I for intermediate resistance phenotype

Seventy seven de novo *gyrA* sequences of CC4821 *N.meningitidis* were analyzed in the context of 149 publicly available *gyrA* sequences (listed in Additional Table 1). A neighbor joining phylogenetic tree was constructed with the resulting dataset of 226 *gyrA* nucleotide sequences (Fig. 1). A similar tree was obtained using maximum likelihood (ML) method (Additional Figure 1). The nucleotide sequences were significantly divergent with an overall p-distance of 0.045 (Table 1). An overview of the tree showed that the sequences from CC4821 *N.meningitidis* strains (in red in Fig. 1) were found across the tree demonstrating that *gyrA* gene was relatively divergent within these strains.

**Table 1:** Summary of the phylogenetic analysis

Group	Within p-dist^a^	Number of sequences	Specific aa.	Species	Collection country	Year of collection	ST	CC	Serogroup	CIP resistance phenotype ^**f**^
Overall	0.045	226				1931–2017				
Outlier	0.143 ^c,d^	15	L123D; A124G; S139A; V269I; I402M; N411D; M443A; S504A	*Eikenella corrodens* (2); *N.animaloris* (2); *N.elongata* (2); *N.weaveri* (2); *N.animalis*; *N.canis*; *N.elongata subspecies glycolytica*; *N.mucosa*; *N.polysaccharea*; *N.sicca*; *N.zoodegmatis*	USA (2); South Korea (1); Norway (1); Germany (1); France (1); Sweden (1)	1954–2017	ST3557; ST3706; ST9346; ST9806			
1	0.06	2	E441H; K455H; S488E; G494S; I509V; E518A; E519D	*N.meningitidis*; *N.subflava*	China;?	2005–2009	ST4832	CC4821(1)	C (1)	I (1)
2	0.017	2	0	*N.meningitidis*	China	2007–2011	ST9477; ST5636	CC4821; UA	B	R (1)
3	0.032 ^b^	1	0	*N.meningitidis*	China	2009	ST12300	CC4821	B	I (1)
4	0.025 ^b^	1	0	*N.meningitidis*	China	2006	ST5751	UA	B	R
5	0.04	26	0	*N.meningitidis* (20); *N.lactamica* (5)	China (13); UK (1); USA (1)	1992–2014	ST5473 (3); ST3493; ST8241; ST4821; ST12316; ST162; ST6930; ST8815; ST5662; ST3436; ST2146; ST8491; ST8920	CC4821 (9); UA (2); CC162; CC32; CC198; CC613	B (7); C (4); W (2); NG	R (11); I (2); S (1)
6	0.03	27	0	*N.meningitidis* (26); *N.cinerea*	China	2005–2014	ST7 (8); ST9454 (2); ST3200 (2); ST5798; ST8241; ST10235; ST5664; ST10446; ST5586; ST5082; ST5084; ST7962; ST6933	CC5 (10); CC4821 (8); UA (2); CC77; CC174	A (9); B (7); NG (2); E(2); C; X	R (19); I (2)
7	0.002	12	D210K, E456K, E483K, V486I, N836S, D917G	*N.gonorrhoeae*	Canada (2); USA (2); Japan (1)	2010–2015	ST1901 (2); ST1903 (2); ST1928; ST7363; ST7367; ST8122; ST8127; ST12536; ST1902			R (5); S (4)
8	0.003 ^e^	140	0	*N.meningitidis*	China (125); USA (5); UK (4); Germany (4); Norway (1); France (1); Iceland (1); Benin (1); Gambia (1); South Africa (1); Burkina Faso (1); Canada (1)	1931–2016	ST4821 (35); ST5 (11); ST11 (5); ST3 (5); ST7 (5); ST3200 (3); ST6933 (2); ST10737 (2); ST32 (2); ST4 (2); ST9454 (2); ST4831 (2); ST5586 (2); ST10; ST10474; ST10607; ST11920; ST12274; ST12276; ST12295; ST12311; ST12314; ST12767; ST12790; ST136; ST177; ST213; ST2146; ST23; ST269; ST2875; ST2881; ST334; ST3436; ST41; ST43; ST4367; ST461; ST4820; ST4896; ST4897; ST4980; ST5242; ST53; ST5464; ST5555; ST5586; ST5610; ST5614; ST5615; ST5632; ST5663; ST5789; ST5798; ST5863; ST5944; ST658; ST6928; ST7303; ST74; ST8445; ST845; ST8789; ST8798; ST9455; ST9456; ST9754; ST9792; ST9936;	CC4821 (68); CC5 (16); CC1 (8); UA (8); CC11 (6); CC174 (3); CC32 (3); CC41/44 (3); CC4 (2); CC175 (2); CC103; CC18; CC181; CC198; CC213; CC23; CC269; CC334; CC364; CC4240/6688; CC461; CC53; CC5615; CC8	C (43); B (41); A (26); W (8); E (2); NG (1); X (2); Y (2); cnl; I	R (28); I(17); S (81)

a- Within p-distance except except ^b^

b- Lowest pairwise p-distance

c- The lowest p-distance between the outlier group and the other 8 groups is 0.16

d- The p-distance within all the sequences except group 8 (86 sequences) is 0.09

e- The p-distance between group 8 and the remaining 86 sequences is 0.066

f- CIP resistance phenotype: R: Resistance; I: Intermediate; S: Susceptible

Among the 226 analyzed sequences, nearly 62% of the sequences (140) were found on the top of the tree, with no significant bootstrap value (Fig. 1). These sequences were highly homogeneous, with a p-distance of 0.003 (Table 1). The remaining 86 sequences were more divergent, with a p-distance of 0.066 relative to sequences grouped on the top of the tree. Most of the nodes concerning these 86 sequences featured a bootstrap value > 70%. Moreover, the p-distance within this group of 86 sequences was 0.09 demonstrating that these sequences were highly divergent between each other. As the major nodes of the tree featured a bootstrap value > 80%, we decided to arbitrarily assign sequences to 9 different genetic groups (Fig. 1; Table 1). These 9 groups were also found in the ML tree (Additional Figure 1). The *gyrA* sequences from CC4821 strains were found in 6 of these genetic groups namely group 1, 2, 3, 5, 6 and 8.

Group 1 consisted of 2 sequences, *N.meningitidis*-MK930374–100514-ST4832-CC4821-C-China-2005 and *N.subflava*-CP031251.1-M18660----2009. This group was supported by a bootstrap of 100% suggesting that these 2 sequences were very different from the rest of the sequences. The sequences shared 7 amino changes (Table 1, Additional Table 2). However, the long branch corresponding to *N.subflava*-CP031251.1-M18660----2009 sequence suggested that this sequence was also significantly divergent from *N.meningitidis*-MK930374–100514-ST4832-CC4821-C-China-2005 and this was confirmed by a p-distance of 0.06 between these 2 sequences (Table 1).

Group 2 consisted of 2 sequences from *N.meningitidis* strains collected in Gansu and Guangxi province in China in 2007 and 2011, namely *N.meningitidis*-KF733178-G1-ST5636-UA-B-China-2007-R and *N.meningitidis*-MK930446-GX34173-ST9477-CC4821-B-China-2011.

Group 3 consisted of a single sequence, namely, *N.meningitidis*-MK930402–231003-ST12300-CC4821-B-China-2009. The node corresponding to this sequence was supported by a bootstrap value of 80%. Furthermore, the lowest pairwise p-distance between this sequence and the other analyzed sequences was 0.032 demonstrating that this sequence was significantly divergent compared to the rest of the analyzed sequences (Table 1).

Group 5 consisted of 26 sequences, mainly from *N.meningitidis* including 9 of CC4821. A subgroup of 7 sequences supported by a bootstrap of 100% featured 5 sequences from *N.lactamica*. Interestingly, *N.meningitidis*-MK930398–140901-ST8241-CC4821-B-China-2009 clustered with *N.lactamica* sequences with a bootstrap of 100% and a long branch.

Group 6 consisted of 27 sequences from *N.meningitidis* (8 of CC4821) and 1 sequence from *N. cinerea*, namely *N.cinerea*-LS483369.1-NCTC10294, which shared a node with *N.meningitidis*-KF733132–59-ST7962-CC77-NG-China-2009-R. However, the *N.cinerea* sequence featured a long branch suggesting a significant divergence compared to the *N.meningitidis* sequence. Based on the available data, the sequences from group 6 appeared to be from strains that were resistant to CIP. However, no unique amino acid substitution was shared by these strains suggesting that there was no common marker for the resistance phenotype of these strains (Additional Table 2).

Group 8 contained most of the *N.meningitidis* sequences (64%) analyzed in this report. The strains were collected in the last 88 years in 13 different countries from 4 continents. Despite the significant time span and geographic spread, these sequences were highly conserved with a p-distance of 0.003. Moreover, these sequences were from strains of 68 STs, 24 CCs, 9 serogroups, including the reference strain 053442. Overall, these observations showed that *gyrA* gene was highly conserved among most *N.meningitidis* strains despite different genetic characteristics, geographic locations or collection time.

### Analysis of the divergence within the GyrA protein

**Fig. 2:**
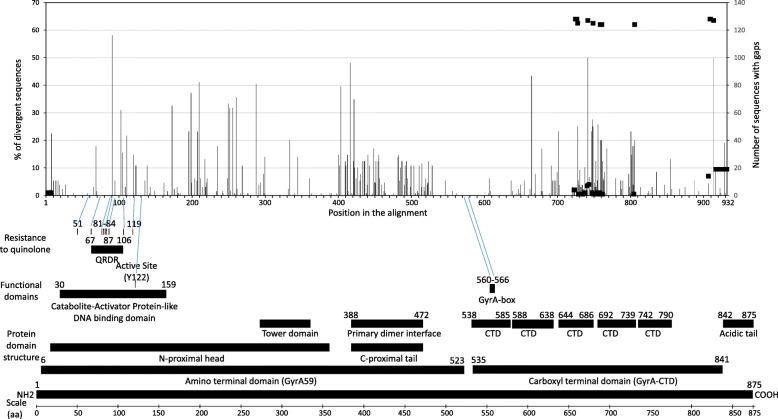
Amino acid divergence among the GyrA protein based on 129 unique sequences. The positions within the 932 amino acid long alignment are indicated on the X axis. The percentage of sequences featuring a particular divergent position is indicated on the Y axis (left side). For example, 58% of the sequences feature a mutation at position 91. The divergence plot was generated from the amino acid difference table shown in Additional Table 2. The alignment features 10 gaps and the number of sequences featuring gaps is indicated on the right axis and shown as a black square. A map of *E.coli* GyrA protein featuring structural and functional domains is shown at the bottom for comparison. The map was generated based on the following references [10, 12, 13]. The GyrA protein sequence of *E.coli* and *N.meningitidis* reference strain 053442 (GB-ID CP000381) were compared (Additional Table 4). The known CIP-resistant site in *E.coli* are shown and corresponding positions in *N.meningitidis* are indicated with a blue line. It is worth noting that among the 8 resistant sites reported in *E.coli*, only positions 83 and 87 are divergent in *N.meningitidis* (sites 91 and 95)

The amino acid divergence within the GyrA protein was analyzed among 129 unique sequences (Additional Table 2). Two hundred fifty-seven divergent positions were identified among the 931 amino acid featured in the alignment (Fig. 2). Even though these sites were found across the protein, the distribution of the divergence did not appear to be random. Indeed, two regions were highly conserved, from positions 530 to 620, and a smaller region between 300 and 330. According to the protein from *E.coli*, the first region corresponds to the end of the amino terminal domain and the beginning of the carboxy terminal domain. The second region corresponds to the tower domain of the protein based on the 3D model structure (Fig. 2).

Among the 257 divergent positions, none were shared by all the analyzed *gyrA* sequences of CC4821 strains. Five sites (91, 417, 665, 210 and 288) were highly divergent, 40% or more of the 129 sequences were mutated at these positions. For example, 48% of the 129 sequences featured a mutated residue at position 417 (Fig. 2). One position (91) appeared to be linked to CIP resistance, all the strains that were not sensitive to CIP were mutated at this position, featuring either an I or an F or a V (Additional Table 2).

### Identification of potential resistance markers to CIP

Among the 226 analyzed sequences, 174 were from strains tested for resistance to CIP (Additional Table 1). Sixty-seven strains were tested for this study. As mentioned above, all the strains that were not sensitive to CIP (either with a resistant phenotype (R in the tree) or intermediate phenotype (I in the tree)) were mutated at position 91. That showed that a mutation at position 91 was linked to resistance mechanism. Among the 67 strains tested in this study, 49 were mutated at position 91 but 23 of these strains had an intermediated resistance phenotype. This suggested that other positions could be involved in the resistance mechanism. In order to identify additional potential markers for resistance, the 226 strains were further analyzed at each mutated position. A change that would be found in resistant strains (including intermediate resistance phenotype) but not found in any sensitive strains would qualify. However, to increase the stringency of the analysis, a mutation found in only one strain would not be considered. Altogether, 33 sites were identified (Additional Table 3; left side of Additional Table 4). For example, H8N was found in 18 resistant strains (including 2 with intermediate phenotype) but not featured in any sensitive strains. All 226 strains were analyzed for these 33 positions. Once again, to increase the stringency of the analysis, a mutation found in at least one sensitive strain would be discarded. Thus, the mutation D95N found in resistant and sensitive strains was not further considered. Altogether, 39 mutations (some at the same position like position 91) were analyzed (in green on the left side of Additional Table 4). A mutation profile was built for the 128 strains featuring at least one mutation of interest (Additional Table 3). Forty-six different profiles were identified, meaning that there were 46 combinations of these 39 mutations among all the analyzed strains (right side of Additional Table 4). Sixteen of the 46 mutation profiles concerned CC4821 strains (numbers in red in Additional Table 4). Twenty-six profiles concerned strains that were known to be CIP resistant (strain names in blue font in Additional Table 4). Among the 39 potential resistant markers, mutations N103D and T91I were the most shared in the profiles with 29 and 27 appearances respectively. However, it is worth noting that other mutations were also well represented like H8N, I111V, E793Q and A679S with 23, 21, 18 and 17 appearances respectively. It is also worth noting that 45% of the resistant strains (58 out of 128) featured only the mutation T91I. As resistance markers were initially described in *E.coli*, a comparison between *gyrA* sequences of *E.coli* and the reference strain *N.meningitidis* 53,442 was necessary in order to check the position of these markers in *E.coli* sequence (Additional Table 5).

### Recombination within *gyrA* gene between *N. spp*.

**Fig. 3:**
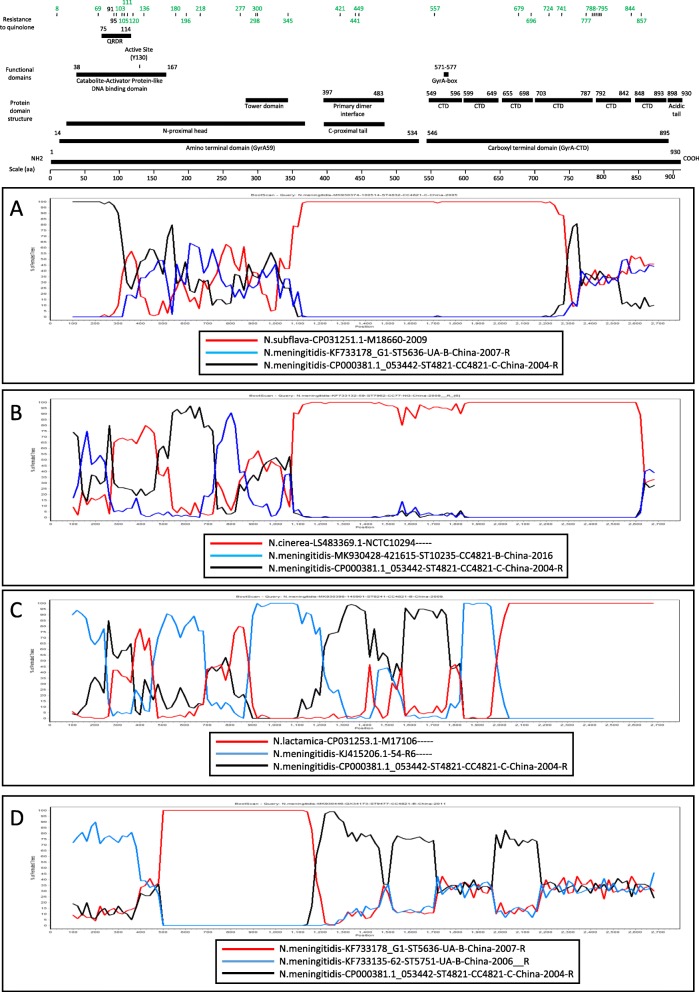
Potential recombination events inter- and intra-species among *Neisseria* strains. **a**. Recombination between *N.subflava* and *N.meningitidis*. A BootScan plot was generated in SimPlot with *N.meningitidis*-MK930374–100514-ST4832-CC4821-C-China-2005 as a query. **b**. Recombination between *N.cinerea* and *N.meningitidis*. A BootScan plot was generated in SimPlot with *N.meningitidis*-KF733132–59-ST7962-CC77-NG-China-2009-R as a query. **c**. Recombination between *N.lactamica* and *N.meningitidis.* A BootScan plot was generated in SimPlot with *N.meningitidis*-MK930398–140901-ST8241-CC4821-B-China-2009 as a query. ***d****.* Recombination between *N.meningitidis* strains*.* A BootScan plot was generated in SimPlot with *N.meningitidis*-MK930446-GX34173-ST9477-CC4821-B-China-2011 as a query. The strain that is predicted to contribute the most in the recombination process (backbone) is shown as a red line. The *N.meningitidis* reference strain 053442 (GB ID CP000381) is shown as a black line. A functional map for the reference strain 053442 is shown above based on the *E.coli* functional map shown in Fig. 2

The phylogenetic analysis identified potential recombinants. For example, the group 1 was of particular interest, concerning *N.meningitidis*-MK930374–100514-ST4832-CC4821-C-China-2005 and *N.subflava*-CP031251.1-M18660. These 2 sequences shared 7 residue changes, not seen in other strains. Furthermore, 5 of these changes were seen within 30 amino acids (Table 1). Finally, amino acid changes observed in one strain were not seen on the other strain, like position 740 and 750. All these observations suggested a recombination between these 2 strains which was confirmed by a BootScan analysis (Fig. 3a). Three other potential recombinations were confirmed by BootScan. Fig. 3b described a recombination between a CC4821 strain (likely either *N.meningitidis*-MK930428–421615-ST10235-CC4821-B-China-2016 or *N.meningitidis*-CP000381.1–053442-ST4821-CC4821-C-China-2004_R) and *N.cinerea*-LS483369.1-NCTC10294 -----. Fig. 3c featured multiple recombination events between *N.lactamica*-CP031253.1-M17106----- and two *N.meningitidis* strains, *N.meningitidis*-KJ415206.1–54-R6----- and *N.meningitidis*-CP000381.1–53,442-ST4821-CC4821-C-China-2004_R. Finally, a recombination between *N.meningitidis* strains was featured in Fig. 3d. Altogether, the recombination analysis showed that the *gyrA* gene of *Neisseria* strains is highly prone to recombination.

## Discussion

CC4821 *N.meningitidis* strains are endemic in China. CC4821 strains have been first detected in 2003 in Anhui province, China. Since then, CC4821 strains have been detected in more than 20 provinces in China and have been causing deaths and serious disease burden [4, 5]. CC4821 strains feature a high rate of CIP resistance, estimated at more than 50% according to Zhu et al., compared to what is observed in other countries, for example 0.58% in Canada or 0.23% in Italy [8, 14, 15]. A recent study concerned the evolution of the *gyrA* gene from CC5 strains from China. It showed 2 main lineages depending on the CIP resistance phenotype. It also showed a quasi-clonal evolution of the strains [8]. The present study on CC4821 strains showed a very different picture. First, more than half of the strains had a conserved *gyrA* gene independent on their CIP resistance phenotype. Second, multiple *gyrA* lineages can be identified and no link to CIP resistance can be identified. Finally, *gyrA* evolution does not appear to be linked to the evolution of any clonal complex. The discrepancy between the *gyrA* evolution of CC4821 and CC5 strains might be due to the fact that CC4821 strains are spread throughout the country, therefore increasing the chance for genetic exchange with other *Neisseria* strains.

CIP is one of the most used of the third generation quinolone antibiotics. It has been recommended for the treatment and prevention of IMD worldwide [16]. Studies in *E.coli* demonstrated that CIP interact with a region called Quinolone Resistance Determining Region located at positions 67–106. Six sites have been reported to be involved in resistance mechanism, namely 67, 81, 83, 84, 87 and 106 [11]. A region similar to QRDR has been mapped in *Neisseria gyrA* counterpart, 75 to 114. However, as shown in the present study, only the position 91 (equivalent to 83 in *E.coli*) appears to be linked to a CIP resistance phenotype. Furthermore, the present study identified strains baring the mutation T91I but featuring an intermediate resistance phenotype. Altogether, this suggests that *Neisseria* might have a different resistance mechanism. An in silico analysis presented in the current study identified 31 positions potentially involved in a resistance mechanism. They are located outside the so-called QRDR. Interestingly, PubMLST, which is a tool to analyze *Neisseria* strains, is using a 525 nt long region (partially covering the 160 nt of QRDR) to identify *gyrA* allele suggesting that sites outside QRDR are likely to be involved in the resistance mechanism. Further analysis would be necessary to assess the role of these positions in the resistance mechanism. Unfortunately, as far as we know, the 3D structure model does not feature some of these sites as it was generated with the GyrA59 region of the protein corresponding to the amino terminal domain of the protein [12].

Recombination between the genomes of *Neisseria* species have been previously reported. For example, Wu et al. reported a likely recombination between the genome of *N.lactamica* and *N.meningitidis* strains [17].The present study reported additional recombination events between the *gyrA* gene of *Neisseria* species. Recombination would mean that the bacteria have been replicating at the same time and same location. Among the reported recombinations, a recombination within *gyrA* gene between *N.meningitidis* and *N.subflava* was identified. Unfortunately, the metadata concerning the *N.subflava* strain was very limited, only the collection year was identified after contacting the scientists who reported the genome sequence in GenBank. Knowing the country of collection would help to understand a potential recombination with *N.meningitidis* strain collected in China. Recombination between species as well as intra-species is likely to allow the strains to acquire new resistant phenotype however further studies would be necessary to better assess the acquisition of the resistance phenotype.

## Conclusions

A phylogenetic analysis of *gyrA* gene from CC4821 *N.meningitidis* strains in the context of other *Neisseria* species showed that *gyrA* gene is well conserved among most CC4821 strains. However, significant divergence is observed in a few strains. GyrA gene evolution does not appear to be linked to CIP resistance phenotype. An in silico analysis of amino acid mutations within GyrA protein showed that only mutations at positions 91 were linked to a CIP resistance phenotype. The analysis also suggested that other sites outside the so-called QRDR could be involved in the resistance mechanism. Recombination inter- and intra-species could explain how strains can acquire mutations leading to various resistance phenotype. Analyzing the evolution of *gyrA* gene is critical to monitor the resistance to quinolone and the acquisition of new resistance markers. Such studies are necessary for the control of the meningococcal disease and the development of new drugs targeting DNA gyrase.

## Methods

### Bacterial strains

More than 4,000 *N.meningitidis* isolates have been collected throughout China from IMD patients as well as close contacts and asymptomatic carriers by our laboratory since 1960s. The bacterial strains were propagated on single Petri dish containing Difco™ Columbia Blood Agar Base with 5% Sheep Blood in a 5% CO_2_ atmosphere at 37 °C for 18 h. Single colonies were lysed and tested by PCR for the meningococcal-specific contact-regulated gene A (*crgA*) in order to identify bacterial species [18]. Strain serogroups were determined by slide agglutination with rabbit antisera specific to each serogroup (BD Difco). Genomic DNA was extracted using the Wizard Genomic DNA Purification Kit (Promega, Madison, WI, USA) according to the manufacturer’s instructions. MLST was performed according to the description of Maiden et al. [3]. Briefly, 7 genes (*abcZ*, *adk*, *aroE*, *fumC*, *gdh*, *pdhC* and *pgm*) were amplified and sequenced. STs and CCs were subsequently assigned by querying the sequence database available at http://pubmlst.org/. Seventy-seven CC4821 isolates were selected as representative isolates based on the typing results (ST and serogroup), isolation year (1978–2016) and sample source (IMD patients, close contacts, or asymptomatic carriers) for in-depth phylogenetic analysis. The *gyrA* gene was sequenced by PCR using the primer pair *gyrA*-F, 5′-GTTCCGCGTCAAAATATGCT-3′ and *gyrA*-2844R, 5′-GACTATAATCCGCTATATTGT-3′ generating a 2905 bp long amplicon containing the 2751 nt of *gyrA* gene [8, 19]. Seventy-seven complete gene sequences were generated and submitted to GenBank (MK930374-MK930450) (shown in yellow in Additional Table 1).

### Dataset

The *gyrA* gene from the reference strain *N.meningitidis* 053442 (CP000381.1) was used to query the GenBank database and 556 additional *gyrA* gene sequences were selected from the BLAST output. Identical sequences were deleted unless the strains did not share the same CC, ST or serogroup. Sequences less than 90% of full length (2905 nt) were discarded. The remaining 149 sequences were combined with the 77 sequences generated in this study. The distribution in terms of species, country of collection and year of collection is shown in Additional Figure 2. Distribution in terms of ST, CC, serogroup as well as CIP resistance phenotype is shown in Additional Figure 3,

### Sequence analysis

Two hundred twenty six *gyrA* nucleotide sequences were aligned using Mega 6 and a neighbor joining phylogenetic tree was generated using the maximum composite likelihood nucleotide model [20, 21]. Phylogenetic inference was tested with 1000 bootstraps [22]. Nodes with bootstrap value > 70% were indicated. Average genetic p-distance within genetic groups as well as between genetic groups and pairwise distances were computed in Mega. Amino acid divergence was analyzed in Mega and processed in Excel. Potential recombination events were analyzed with the SimPlot software [23].

### CIP resistance test

*N.meningitidis* strains were incubated at 35 °C, 5% CO_2_ in chocolate agar (Detgerm, China) for 20–24 h. A single colony was selected and grown to reach a 0.5 McFarland standard. *Escherichia coli* ATCC 25922 strain was used as a quality control strain. The bacterial suspension was evenly spread on Mueller-Hinton blood plate (OXOID, US). A CIP Epsilometer-test strip (Liofilchem, Italy) was then attached to the plate. The Minimal Inhibitory Concentration (MIC) was read after incubation at 35 °C, 5% CO2 for 18–20 h. The CIP resistance test was interpreted as established by Clinical and Laboratory Standards Institute (CLSI): S(susceptible) ≤ 0.03 μg/mL, I(intermediate) = 0.06 μg/mL, R(resistant) ≥ 0.12 μg/mL [24].

## Supplementary information

**Additional file1 Figure S1.** Maximum likelihood phylogenetic tree of 226 *gyrA* gene sequences from *Neisseria* strains. The tree was annotated according to Fig. 1.

**Additional file 2 Figure S2.** Distribution of species, collection country and year of collection for the 226 *gyrA* gene sequences analyzed in this study. A. Distribution of species. The number of strains for each species is plotted. The dataset contains 192 *N.meningitidis* strains and 12 *N.gonorrhoeae* strains. The species are organized based on their host, human specific first. *Eikenella* belongs to the *Neisseria* genus. B. Distribution based on collection country. The dataset contains 146 strains from China. C. Distribution based on collection year.

**Additional file 3 Figure S3.** Distribution of ST, CC, serogroup as well as CIP resistance phenotype of the 192 *N.meningitidis* strains analyzed in this study. A. Distribution of ST among the 88 CC4821 strains. B. Distributions of CC and ST of 86 strains which do not belong to CC4821 and are represented by 2 or more STs in the dataset. ND; Not determined. Strains with no ST are indicated with “-“. C. Distributions of CC and ST of 18 strains represented by only 1 ST in the dataset. D. Distribution of serogroup among the 192 *N.meningitidis* strains analyzed in this study. Serogroup information was not available for 26 strains. The CIP resistance phenotype is color coded, orange bar for sensitive strains and grey bar for resistance strains. The strains for which the CIP resistance has not been tested are indicated with a blue bar. Intermediate phenotype was considered as resistance in the figure.

**Additional file 4 Table S1.** List of the 226 analyzed strains.

**Additional file 5 Table S2.** Amino acid difference among the 226 GyrA protein sequences.

**Additional file 6 Table S3.** Potential CIP resistance markers among the 226 GyrA protein sequences analyzed in this study.

**Additional file 7 Table S4.** Putative resistance markers based on the analysis of 226 GyrA protein sequences.

**Additional file 8 Table S5.** Alignment of GyrA protein sequence from *E.coli* K12 and *N.meningitidis* reference strain 053442.

## Data Availability

All data generated or analyzed during this study are included in this published article and its supplementary information files. Seventy-seven complete gene sequences were generated and submitted to GenBank (MK930374-MK930450).

## Abbreviations

CC: Clonal Complex
CIP: Ciprofloxacin
*gyrA*: Gyrase subunit A
IMD: Invasive Meningococcal Disease
MLST: Multi-Locus Sequence Typing
*N. meningitidis*: *Neisseria meningitidis*
QRDR: Quinolone Resistance-Determining Region
ST: Sequence Types
