# Supplementary material for: Evolutionary analysis of gyrA gene from Neisseria meningitidis bacterial strains of clonal complex 4821 collected in China between 1978 and 2016
Source: BMC Microbiol. 2020 Mar 30;20:71. doi: 10.1186/s12866-020-01751-5 (PMC7106703; doi:10.1186/s12866-020-01751-5)
Supplement: Supplementary file 1 — Additional file1 Figure S1. Maximum likelihood phylogenetic tree of 226 gyrA gene sequences from Neisseria strains. The tree was annotated according to Fig. 1. [file 12866_2020_1751_MOESM1_ESM.pptx]

## Slide 1
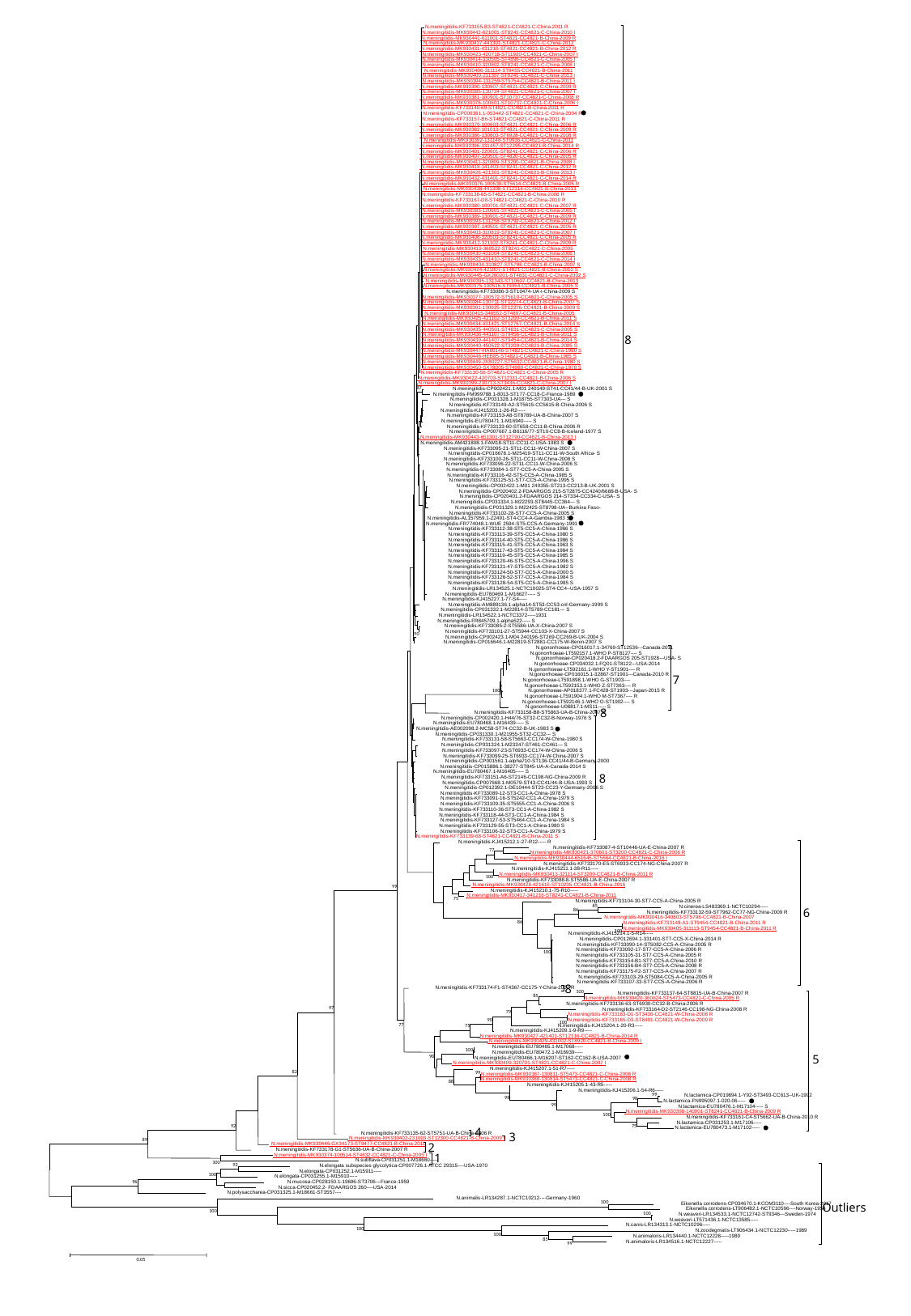

N.meningitidis-KF733155-B3-ST4821-CC4821-C-China-2011 R
 N.meningitidis-MK930442-621001-ST8241-CC4821-C-China-2010 I
 N.meningitidis-MK930441-611001-ST4821-CC4821-B-China-2009 R
 N.meningitidis-MK930437-441301-ST4821-CC4821-C-China-2012
 N.meningitidis-MK930431-431210-ST4821-CC4821-B-China-2012 R
 N.meningitidis-MK930423-420718-ST11920-CC4821-C-China-2007 I
 N.meningitidis-MK930414-330505-ST4896-CC4821-C-China-2005 I
 N.meningitidis-MK930410-320802-ST8241-CC4821-C-China-2008 I
 N.meningitidis-MK930406-311114-ST9455-CC4821-B-China-2011
 N.meningitidis-MK930400-211307-ST8241-CC4821-C-China-2013 I
 N.meningitidis-MK930394-131259-ST9754-CC4821-B-China-2011 I
 N.meningitidis-MK930390-130907-ST4821-CC4821-C-China-2009 R
 N.meningitidis-MK930385-130724-ST4821-CC4821-C-China-2007 I
 N.meningitidis-MK930381-100901-ST10737-CC4821-C-China-2008 R
 N.meningitidis-MK930378-100601-ST10737-CC4821-C-China-2006 I
 N.meningitidis-KF733140-69-ST4821-CC4821-B-China-2011 R
 N.meningitidis-CP000381.1-053442-ST4821-CC4821-C-China-2004 R
 N.meningitidis-KF733157-B6-ST4821-CC4821-C-China-2011 R
 N.meningitidis-MK930379-100603-ST4821-CC4821-C-China-2006 R
 N.meningitidis-MK930382-101013-ST4821-CC4821-C-China-2009 R
 N.meningitidis-MK930386-130803-ST6928-CC4821-C-China-2008 R
 N.meningitidis-MK930392-131148-ST9936-CC4821-C-China-2011
 N.meningitidis-MK930396-131457-ST12295-CC4821-B-China-2014 R
 N.meningitidis-MK930401-220601-ST8241-CC4821-C-China-2006 R
 N.meningitidis-MK930407-320501-ST4820-CC4821-C-China-2005 R
 N.meningitidis-MK930411-320809-ST3200-CC4821-B-China-2008 I
 N.meningitidis-MK930418-341403-ST8241-CC4821-C-China-2012 R
 N.meningitidis-MK930426-421301-ST8241-CC4821-B-China-2013 I
 N.meningitidis-MK930432-431401-ST8241-CC4821-C-China-2014 R
 N.meningitidis-MK930376-100530-ST5614-CC4821-B-China-2005 R
 N.meningitidis-MK930438-441308-ST12314-CC4821-B-China-2013
 N.meningitidis-KF733138-65-ST4821-CC4821-B-China-2008 R
 N.meningitidis-KF733167-D8-ST4821-CC4821-C-China-2010 R
 N.meningitidis-MK930380-100701-ST4821-CC4821-C-China-2007 R
 N.meningitidis-MK930383-120601-ST4821-CC4821-C-China-2005 I
 N.meningitidis-MK930389-130901-ST4821-CC4821-C-China-2009 R
 N.meningitidis-MK930393-131258-ST9792-CC4821-C-China-2012 I
 N.meningitidis-MK930397-140501-ST4821-CC4821-C-China-2005 R
 N.meningitidis-MK930403-310819-ST8241-CC4821-C-China-2007 I
 N.meningitidis-MK930408-320503-ST8241-CC4821-C-China-2005 R
 N.meningitidis-MK930412-321102-ST8241-CC4821-C-China-2009 R
 N.meningitidis-MK930419-360522-ST8241-CC4821-C-China-2005
 N.meningitidis-MK930430-431004-ST8241-CC4821-C-China-2008 I
 N.meningitidis-MK930433-431410-ST8241-CC4821-C-China-2014 I
 N.meningitidis-MK930404-310827-ST5798-CC4821-B-China-2007 S
 N.meningitidis-MK930424-421007-ST4821-CC4821-B-China-2010 S
 N.meningitidis-MK930445-GX200201-ST4831-CC4821-C-China-2002 S
 N.meningitidis-MK930395-131343-ST10607-CC4821-B-China-2013
 N.meningitidis-MK930375-100516-ST9454-CC4821-B-China-2005 S
 N.meningitidis-KF733086-3-ST10474-UA-I-China-2009 S
 N.meningitidis-MK930377-100572-ST5610-CC4821-C-China-2005 S
 N.meningitidis-MK930384-130711-ST12274-CC4821-B-China-2007 S
 N.meningitidis-MK930391-130925-ST12276-CC4821-B-China-2009 S
 N.meningitidis-MK930415-340552-ST4897-CC4821-B-China-2005
 N.meningitidis-MK930425-421102-ST3200-CC4821-B-China-2011 S
 N.meningitidis-MK930434-431421-ST12767-CC4821-B-China-2014 S
 N.meningitidis-MK930435-440501-ST4831-CC4821-C-China-2005 S
 N.meningitidis-MK930436-441107-ST9456-CC4821-B-China-2011 S
 N.meningitidis-MK930439-441407-ST9454-CC4821-B-China-2014 S
 N.meningitidis-MK930440-450522-ST3200-CC4821-B-China-2005 S
 N.meningitidis-MK930447-HA80146-ST4821-CC4821-C-China-1980 S
 N.meningitidis-MK930448-HEB85-ST4821-CC4821-B-China-1985 S
 N.meningitidis-MK930449-JX80227-ST5632-CC4821-B-China-1980 S
 N.meningitidis-MK930450-SX78005-ST4980-CC4821-C-China-1978 S
 N.meningitidis-KF733130-56-ST4821-CC4821-C-China-2005 R
 N.meningitidis-MK930422-420703-ST12311-CC4821-B-China-2006 S
 N.meningitidis-MK930399-210713-ST3436-CC4821-C-China-2007 I
 N.meningitidis-CP002421.1-M01 240149-ST41-CC41/44-B-UK-2001 S
 N.meningitidis-FM999788.1-8013-ST177-CC18-C-France-1989
 N.meningitidis-CP031328.1-M18755-ST7303-UA--- S
 N.meningitidis-KF733149-A2-ST5615-CC5615-B-China-2006 S
 N.meningitidis-KJ415203.1-26-R2-----
 N.meningitidis-KF733153-A8-ST8789-UA-B-China-2007 S
 N.meningitidis-EU780471.1-M16940----- S
 N.meningitidis-KF733133-60-ST658-CC11-B-China-2006 R
 N.meningitidis-CP007667.1-B6116/77-ST10-CC8-B-Iceland-1977 S
 N.meningitidis-MK930443-651301-ST12790-CC4821-B-China-2013 I
 N.meningitidis-AM421808.1-FAM18-ST11-CC11-C-USA-1983 S
 N.meningitidis-KF733095-21-ST11-CC11-W-China-2007 S
 N.meningitidis-CP016678.1-M25419-ST11-CC11-W-South Africa- S
 N.meningitidis-KF733100-26-ST11-CC11-W-China-2008 S
 N.meningitidis-KF733096-22-ST11-CC11-W-China-2006 S
 N.meningitidis-KF733084-1-ST7-CC5-A-China-2005 S
 N.meningitidis-KF733116-42-ST5-CC5-A-China-1985 S
 N.meningitidis-KF733125-51-ST7-CC5-A-China-1995 S
 N.meningitidis-CP002422.1-M01 240355-ST213-CC213-B-UK-2001 S
 N.meningitidis-CP020402.2-FDAARGOS 215-ST2875-CC4240/6688-B-USA- S
 N.meningitidis-CP020401.2-FDAARGOS 214-ST334-CC334-C-USA- S
 N.meningitidis-CP031334.1-M22293-ST8445-CC364--- S
 N.meningitidis-CP031329.1-M22425-ST8798-UA--Burkina Faso-
 N.meningitidis-KF733102-28-ST7-CC5-A-China-2005 S
 N.meningitidis-AL157959.1-Z2491-ST4-CC4-A-Gambia-1983 S
8
 N.meningitidis-FR774048.1-WUE 2594-ST5-CC5-A-Germany-1991
 N.meningitidis-KF733112-38-ST5-CC5-A-China-1966 S
 N.meningitidis-KF733113-39-ST5-CC5-A-China-1980 S
 N.meningitidis-KF733114-40-ST5-CC5-A-China-1986 S
 N.meningitidis-KF733115-41-ST5-CC5-A-China-1963 S
 N.meningitidis-KF733117-43-ST5-CC5-A-China-1984 S
 N.meningitidis-KF733119-45-ST5-CC5-A-China-1985 S
 N.meningitidis-KF733120-46-ST5-CC5-A-China-1996 S
 N.meningitidis-KF733121-47-ST5-CC5-A-China-1982 S
 N.meningitidis-KF733124-50-ST7-CC5-A-China-2000 S
 N.meningitidis-KF733126-52-ST7-CC5-A-China-1984 S
 N.meningitidis-KF733128-54-ST5-CC5-A-China-1985 S
 N.meningitidis-LR134525.1-NCTC10025-ST4-CC4--USA-1957 S
 N.meningitidis-EU780469.1-M16627----- S
 N.meningitidis-KJ415227.1-77-S4-----
 N.meningitidis-AM889136.1-alpha14-ST53-CC53-cnl-Germany-1999 S
 N.meningitidis-CP031332.1-M22814-ST5789-CC181--- S
 N.meningitidis-LR134522.1-NCTC3372-----1931
 N.meningitidis-FR845709.1-alpha522----- S
 N.meningitidis-KF733085-2-ST5586-UA-X-China-2007 S
 N.meningitidis-KF733101-27-ST5944-CC103-X-China-2007 S
 N.meningitidis-CP002423.1-M04 240196-ST269-CC269-B-UK-2004 S
 N.meningitidis-CP016646.1-M22819-ST2881-CC175-W-Benin-2007 S
 N.gonorrhoeae-CP016017.1-34769-ST12536---Canada-2011
 N.gonorrhoeae-LT592157.1-WHO P-ST8127---- S
 N.gonorrhoeae-CP020418.2-FDAARGOS 205-ST1928---USA- S
 N.gonorrhoeae-CP034032.1-FQ01-ST8122---USA-2014
 N.gonorrhoeae-LT592161.1-WHO Y-ST1901---- R
 N.gonorrhoeae-CP016015.1-32867-ST1901---Canada-2010 R
7
 N.gonorrhoeae-LT591898.1-WHO G-ST1903----
 N.gonorrhoeae-LT592153.1-WHO Z-ST7363---- R
 N.gonorrhoeae-AP018377.1-FC428-ST1903---Japan-2015 R
 N.gonorrhoeae-LT591904.1-WHO M-ST7367---- R
 N.gonorrhoeae-LT592146.1-WHO O-ST1902---- S
 N.gonorrhoeae-U08817.1-MS11----- S
8
 N.meningitidis-KF733158-B8-ST5863-UA-B-China-2007 S
 N.meningitidis-CP002420.1-H44/76-ST32-CC32-B-Norway-1976 S
 N.meningitidis-EU780468.1-M16439----- S
 N.meningitidis-AE002098.2-MC58-ST74-CC32-B-UK-1983 S
 N.meningitidis-CP031330.1-M21955-ST32-CC32--- S
 N.meningitidis-KF733131-58-ST5663-CC174-W-China-1980 S
 N.meningitidis-CP031324.1-M23347-ST461-CC461--- S
 N.meningitidis-KF733097-23-ST6933-CC174-W-China-2006 S
 N.meningitidis-KF733099-25-ST6933-CC174-W-China-2007 S
 N.meningitidis-CP001561.1-alpha710-ST136-CC41/44-B-Germany-2000
 N.meningitidis-CP015886.1-38277-ST845-UA-A-Canada-2014 S
 N.meningitidis-EU780467.1-M16405----- S
8
 N.meningitidis-KF733151-A6-ST2146-CC198-NG-China-2009 R
 N.meningitidis-CP007668.1-M0579-ST43-CC41/44-B-USA-1993 S
 N.meningitidis-CP012392.1-DE10444-ST23-CC23-Y-Germany-2006 S
 N.meningitidis-KF733089-12-ST3-CC1-A-China-1978 S
 N.meningitidis-KF733091-16-ST5242-CC1-A-China-1979 S
 N.meningitidis-KF733109-35-ST5555-CC1-A-China-2006 S
 N.meningitidis-KF733110-36-ST3-CC1-A-China-1982 S
 N.meningitidis-KF733118-44-ST3-CC1-A-China-1984 S
 N.meningitidis-KF733127-53-ST5464-CC1-A-China-1984 S
 N.meningitidis-KF733129-55-ST3-CC1-A-China-1980 S
 N.meningitidis-KF733106-32-ST3-CC1-A-China-1979 S
 N.meningitidis-KF733139-68-ST4821-CC4821-B-China-2011 S
 N.meningitidis-KJ415212.1-27-R12----- R
 N.meningitidis-KF733087-4-ST10446-UA-E-China-2007 R
 N.meningitidis-MK930421-370601-ST3200-CC4821-C-China-2006 R
 N.meningitidis-MK930444-651645-ST5664-CC4821-B-China-2016 I
 N.meningitidis-KF733170-E5-ST6933-CC174-NG-China-2007 R
 N.meningitidis-KJ415211.1-38-R11-----
 N.meningitidis-MK930413-321114-ST3200-CC4821-B-China-2011 R
 N.meningitidis-KF733088-8-ST5586-UA-E-China-2007 R
 N.meningitidis-MK930428-421615-ST10235-CC4821-B-China-2016
 N.meningitidis-KJ415210.1-75-R10-----
 N.meningitidis-MK930417-341216-ST8241-CC4821-B-China-2011
87
90
100
 N.meningitidis-KF733104-30-ST7-CC5-A-China-2005 R
 N.cinerea-LS483369.1-NCTC10294-----
6
 N.meningitidis-KF733132-59-ST7962-CC77-NG-China-2009 R
 N.meningitidis-MK930416-340803-ST5798-CC4821-B-China-2007
 N.meningitidis-KF733148-A1-ST9454-CC4821-B-China-2011 R
 N.meningitidis-MK930405-311113-ST9454-CC4821-B-China-2011 R
 N.meningitidis-KJ415214.1-5-R14-----
 N.meningitidis-CP012694.1-331401-ST7-CC5-X-China-2014 R
 N.meningitidis-KF733090-14-ST5082-CC5-A-China-2005 R
 N.meningitidis-KF733092-17-ST7-CC5-A-China-2006 R
 N.meningitidis-KF733105-31-ST7-CC5-A-China-2005 R
 N.meningitidis-KF733154-B1-ST7-CC5-A-China-2010 R
 N.meningitidis-KF733156-B4-ST7-CC5-A-China-2008 R
 N.meningitidis-KF733175-F2-ST7-CC5-A-China-2007 R
 N.meningitidis-KF733103-29-ST5084-CC5-A-China-2005 R
 N.meningitidis-KF733107-33-ST7-CC5-A-China-2006 R
8
 N.meningitidis-KF733174-F1-ST4367-CC175-Y-China-2007 R
 N.meningitidis-KF733137-64-ST8815-UA-B-China-2007 R
 N.meningitidis-MK930420-360624-ST5473-CC4821-C-China-2005 R
 N.meningitidis-KF733136-63-ST6930-CC32-B-China-2006 R
 N.meningitidis-KF733164-D2-ST2146-CC198-NG-China-2008 R
 N.meningitidis-KF733163-D1-ST3436-CC4821-W-China-2008 R
 N.meningitidis-KF733165-D3-ST8491-CC4821-W-China-2009 R
 N.meningitidis-KJ415204.1-20-R3-----
 N.meningitidis-KJ415209.1-9-R9-----
 N.meningitidis-MK930427-421401-ST12316-CC4821-B-China-2014 R
 N.meningitidis-MK930429-431002-ST8920-CC4821-B-China-2009 I
 N.meningitidis-EU780465.1-M17068-----
 N.meningitidis-EU780472.1-M16939-----
5
 N.meningitidis-EU780466.1-M16207-ST162-CC162-B-USA-2007
 N.meningitidis-MK930409-320701-ST4821-CC4821-C-China-2007 I
 N.meningitidis-KJ415207.1-51-R7-----
 N.meningitidis-MK930387-130811-ST5473-CC4821-C-China-2008 R
 N.meningitidis-MK930388-130814-ST5473-CC4821-C-China-2008 R
 N.meningitidis-KJ415205.1-43-R5-----
 N.meningitidis-KJ415206.1-54-R6-----
 N.lactamica-CP019894.1-Y92-ST3493-CC613--UK-1992
 N.lactamica-FN995097.1-020-06-----
 N.lactamica-EU780476.1-M17104----- S
 N.meningitidis-MK930398-140901-ST8241-CC4821-B-China-2009 R
 N.meningitidis-KF733161-C4-ST5662-UA-B-China-2010 R
 N.lactamica-CP031253.1-M17106-----
4
 N.lactamica-EU780473.1-M17102-----
 N.meningitidis-KF733135-62-ST5751-UA-B-China-2006 R
3
 N.meningitidis-MK930402-231003-ST12300-CC4821-B-China-2009 I
2
 N.meningitidis-MK930446-GX34173-ST9477-CC4821-B-China-2011
 N.meningitidis-KF733178-G1-ST5636-UA-B-China-2007 R
1
 N.meningitidis-MK930374-100514-ST4832-CC4821-C-China-2005 I
 N.subflava-CP031251.1-M18660-----
 N.elongata subspecies glycolytica-CP007726.1-ATCC 29315----USA-1970
 N.elongata-CP031252.1-M15911-----
 N.elongata-CP031255.1-M15910-----
 N.mucosa-CP028150.1-19696-ST3706---France-1959
 N.sicca-CP020452.2- FDAARGOS 260----USA-2014
 N.polysaccharea-CP031325.1-M18661-ST3557----
 N.animalis-LR134287.1-NCTC10212----Germany-1960
 Eikenella corrodens-CP034670.1-KCOM3110----South Korea-2017
 Eikenella corrodens-LT906482.1-NCTC10596----Norway-1954
 N.weaveri-LR134533.1-NCTC12742-ST9346---Sweden-1974
 N.weaveri-LT571436.1-NCTC13585-----
 N.canis-LR134313.1-NCTC10296-----
77
100
99
75
85
86
86
100
100
100
85
97
79
90
100
77
75
100
98
82
99
88
99
98
98
99
100
92
75
89
100
92
100
96
100
Outliers
100
100
100
 N.zoodegmatis-LT906434.1-NCTC12230-----1989
100
 N.animaloris-LR134440.1-NCTC12228-----1989
85
 N.animaloris-LR134516.1-NCTC12227-----
99
0.05
