# Supplementary material for: Evolutionary analysis of gyrA gene from Neisseria meningitidis bacterial strains of clonal complex 4821 collected in China between 1978 and 2016
Source: BMC Microbiol. 2020 Mar 30;20:71. doi: 10.1186/s12866-020-01751-5 (PMC7106703; doi:10.1186/s12866-020-01751-5)
Supplement: Supplementary file 2 — Additional file 2 Figure S2. Distribution of species, collection country and year of collection for the 226 gyrA gene sequences analyzed in this study. A. Distribution of species. The number of strains for each species is plotted. The dataset contains 192 N.meningitidis strains and 12 N.gonorrhoeae strains. The species are organized based on their host, human specific first. Eikenella belongs to the Neisseria genus. B. Distribution based on collection country. The dataset contains 146 strains from China. C. Distribution based on collection year. [file 12866_2020_1751_MOESM2_ESM.pptx]

## Slide 1
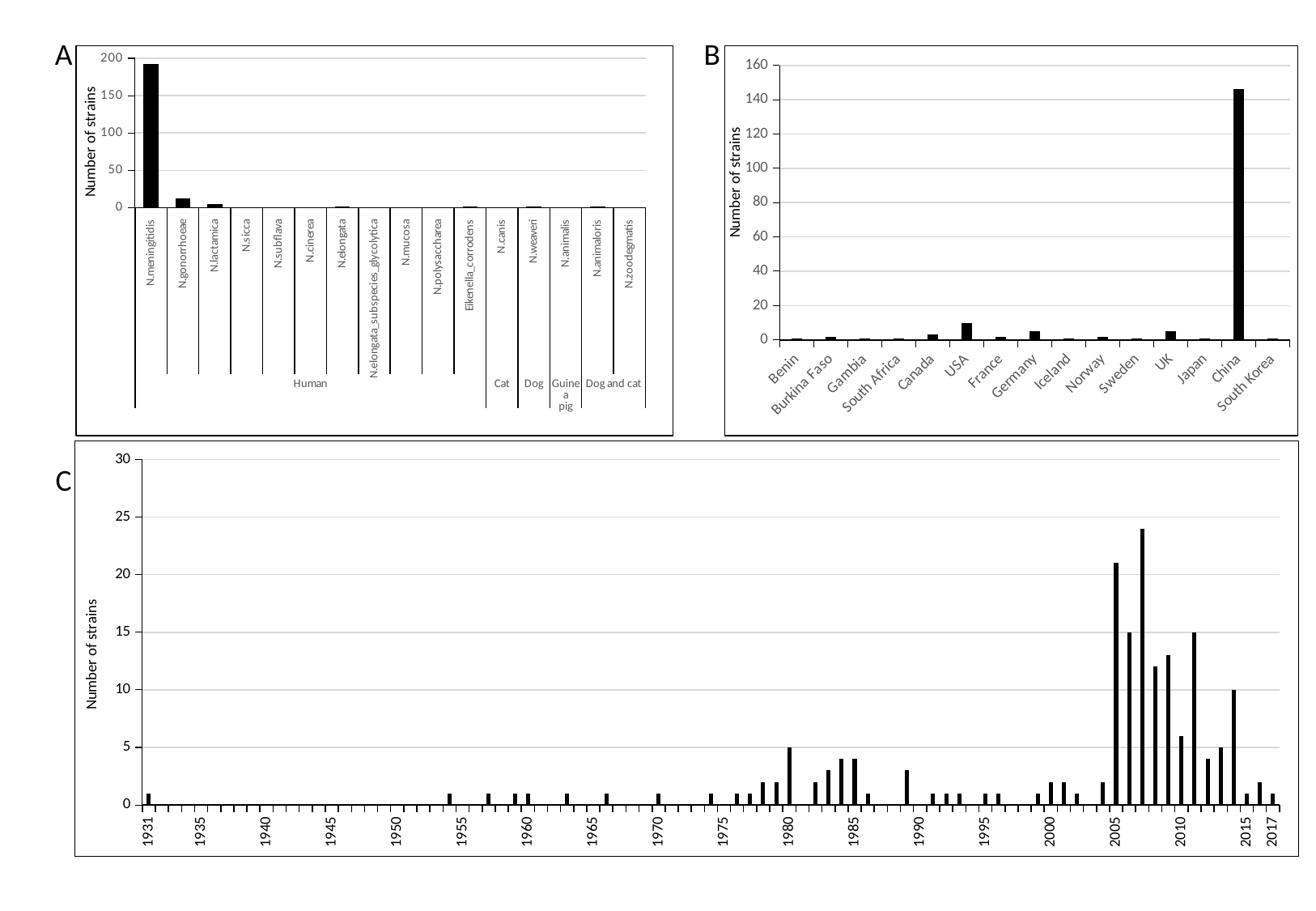

A
B
### Chart
| Category | |
|---|---|
| N.meningitidis | 192.0 |
| N.gonorrhoeae | 12.0 |
| N.lactamica | 5.0 |
| N.sicca | 1.0 |
| N.subflava | 1.0 |
| N.cinerea | 1.0 |
| N.elongata | 2.0 |
| N.elongata_subspecies_glycolytica | 1.0 |
| N.mucosa | 1.0 |
| N.polysaccharea | 1.0 |
| Eikenella_corrodens | 2.0 |
| N.canis | 1.0 |
| N.weaveri | 2.0 |
| N.animalis | 1.0 |
| N.animaloris | 2.0 |
| N.zoodegmatis | 1.0 |
### Chart
| Category | |
|---|---|
| Benin | 1.0 |
| Burkina Faso | 2.0 |
| Gambia | 1.0 |
| South Africa | 1.0 |
| Canada | 3.0 |
| USA | 10.0 |
| France | 2.0 |
| Germany | 5.0 |
| Iceland | 1.0 |
| Norway | 2.0 |
| Sweden | 1.0 |
| UK | 5.0 |
| Japan | 1.0 |
| China | 146.0 |
| South Korea | 1.0 |Number of strains
Number of strains
### Chart
| Category | |
|---|---|
| 1931 | 1.0 |
| | None |
| | None |
| | None |
| 1935 | None |
| | None |
| | None |
| | None |
| | None |
| 1940 | None |
| | None |
| | None |
| | None |
| | None |
| 1945 | None |
| | None |
| | None |
| | None |
| | None |
| 1950 | None |
| | None |
| | None |
| | None |
| | 1.0 |
| 1955 | None |
| | None |
| | 1.0 |
| | None |
| | 1.0 |
| 1960 | 1.0 |
| | None |
| | None |
| | 1.0 |
| | None |
| 1965 | None |
| | 1.0 |
| | None |
| | None |
| | None |
| 1970 | 1.0 |
| | None |
| | None |
| | None |
| | 1.0 |
| 1975 | None |
| | 1.0 |
| | 1.0 |
| | 2.0 |
| | 2.0 |
| 1980 | 5.0 |
| | None |
| | 2.0 |
| | 3.0 |
| | 4.0 |
| 1985 | 4.0 |
| | 1.0 |
| | None |
| | None |
| | 3.0 |
| 1990 | None |
| | 1.0 |
| | 1.0 |
| | 1.0 |
| | None |
| 1995 | 1.0 |
| | 1.0 |
| | None |
| | None |
| | 1.0 |
| 2000 | 2.0 |
| | 2.0 |
| | 1.0 |
| | None |
| | 2.0 |
| 2005 | 21.0 |
| | 15.0 |
| | 24.0 |
| | 12.0 |
| | 13.0 |
| 2010 | 6.0 |
| | 15.0 |
| | 4.0 |
| | 5.0 |
| | 10.0 |
| 2015 | 1.0 |
| | 2.0 |
| 2017 | 1.0 |C
Number of strains
