# Supplementary material for: Evolutionary analysis of gyrA gene from Neisseria meningitidis bacterial strains of clonal complex 4821 collected in China between 1978 and 2016
Source: BMC Microbiol. 2020 Mar 30;20:71. doi: 10.1186/s12866-020-01751-5 (PMC7106703; doi:10.1186/s12866-020-01751-5)
Supplement: Supplementary file 3 — Additional file 3 Figure S3. Distribution of ST, CC, serogroup as well as CIP resistance phenotype of the 192 N.meningitidis strains analyzed in this study. A. Distribution of ST among the 88 CC4821 strains. B. Distributions of CC and ST of 86 strains which do not belong to CC4821 and are represented by 2 or more STs in the dataset. ND; Not determined. Strains with no ST are indicated with “-“. C. Distributions of CC and ST of 18 strains represented by only 1 ST in the dataset. D. Distribution of serogroup among the 192 N.meningitidis strains analyzed in this study. Serogroup information was not available for 26 strains. The CIP resistance phenotype is color coded, orange bar for sensitive strains and grey bar for resistance strains. The strains for which the CIP resistance has not been tested are indicated with a blue bar. Intermediate phenotype was considered as resistance in the figure. [file 12866_2020_1751_MOESM3_ESM.pptx]

## Slide 1
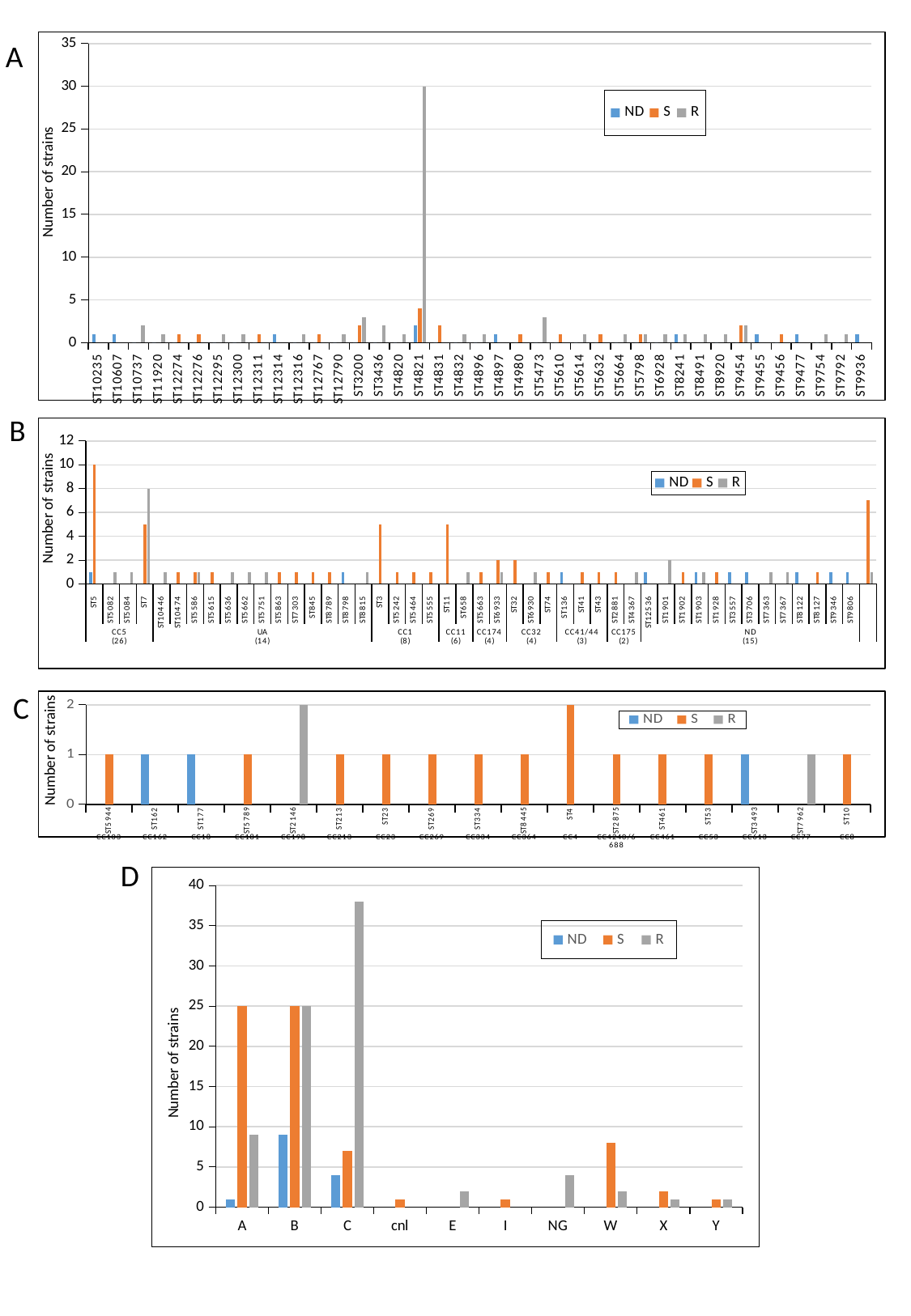

A
### Chart
| Category | | | |
|---|---|---|---|
| ST10235 | 1.0 | None | None |
| ST10607 | 1.0 | None | None |
| ST10737 | 0.0 | None | 2.0 |
| ST11920 | 0.0 | None | 1.0 |
| ST12274 | 0.0 | 1.0 | None |
| ST12276 | 0.0 | 1.0 | None |
| ST12295 | 0.0 | None | 1.0 |
| ST12300 | 0.0 | None | 1.0 |
| ST12311 | 0.0 | 1.0 | None |
| ST12314 | 1.0 | None | None |
| ST12316 | 0.0 | None | 1.0 |
| ST12767 | 0.0 | 1.0 | None |
| ST12790 | 0.0 | None | 1.0 |
| ST3200 | 0.0 | 2.0 | 3.0 |
| ST3436 | 0.0 | None | 2.0 |
| ST4820 | 0.0 | None | 1.0 |
| ST4821 | 2.0 | 4.0 | 30.0 |
| ST4831 | 0.0 | 2.0 | None |
| ST4832 | 0.0 | None | 1.0 |
| ST4896 | 0.0 | None | 1.0 |
| ST4897 | 1.0 | None | None |
| ST4980 | 0.0 | 1.0 | None |
| ST5473 | 0.0 | None | 3.0 |
| ST5610 | 0.0 | 1.0 | None |
| ST5614 | 0.0 | None | 1.0 |
| ST5632 | 0.0 | 1.0 | None |
| ST5664 | 0.0 | None | 1.0 |
| ST5798 | 0.0 | 1.0 | 1.0 |
| ST6928 | 0.0 | None | 1.0 |
| ST8241 | 1.0 | None | 1.0 |
| ST8491 | None | None | 1.0 |
| ST8920 | 0.0 | None | 1.0 |
| ST9454 | 0.0 | 2.0 | 2.0 |
| ST9455 | 1.0 | None | None |
| ST9456 | 0.0 | 1.0 | None |
| ST9477 | 1.0 | None | None |
| ST9754 | 0.0 | None | 1.0 |
| ST9792 | 0.0 | None | 1.0 |
| ST9936 | 1.0 | None | None |Number of strains
B
### Chart
| Category | | | |
|---|---|---|---|
| ST5 | 1.0 | 10.0 | None |
| ST5082 | None | None | 1.0 |
| ST5084 | None | None | 1.0 |
| ST7 | None | 5.0 | 8.0 |
| ST10446 | None | None | 1.0 |
| ST10474 | None | 1.0 | None |
| ST5586 | None | 1.0 | 1.0 |
| ST5615 | None | 1.0 | None |
| ST5636 | None | None | 1.0 |
| ST5662 | None | None | 1.0 |
| ST5751 | None | None | 1.0 |
| ST5863 | None | 1.0 | None |
| ST7303 | None | 1.0 | None |
| ST845 | None | 1.0 | None |
| ST8789 | None | 1.0 | None |
| ST8798 | 1.0 | None | None |
| ST8815 | None | None | 1.0 |
| ST3 | None | 5.0 | None |
| ST5242 | None | 1.0 | None |
| ST5464 | None | 1.0 | None |
| ST5555 | None | 1.0 | None |
| ST11 | None | 5.0 | None |
| ST658 | None | None | 1.0 |
| ST5663 | None | 1.0 | None |
| ST6933 | None | 2.0 | 1.0 |
| ST32 | None | 2.0 | None |
| ST6930 | None | None | 1.0 |
| ST74 | None | 1.0 | None |
| ST136 | 1.0 | None | None |
| ST41 | None | 1.0 | None |
| ST43 | None | 1.0 | None |
| ST2881 | None | 1.0 | None |
| ST4367 | None | None | 1.0 |
| ST12536 | 1.0 | None | None |
| ST1901 | None | None | 2.0 |
| ST1902 | None | 1.0 | None |
| ST1903 | 1.0 | None | 1.0 |
| ST1928 | None | 1.0 | None |
| ST3557 | 1.0 | None | None |
| ST3706 | 1.0 | None | None |
| ST7363 | None | None | 1.0 |
| ST7367 | None | None | 1.0 |
| ST8122 | 1.0 | None | None |
| ST8127 | None | 1.0 | None |
| ST9346 | 1.0 | None | None |
| ST9806 | 1.0 | None | None |Number of strains
C
### Chart
| Category | | | |
|---|---|---|---|
| ST5944 | None | 1.0 | None |
| ST162 | 1.0 | None | None |
| ST177 | 1.0 | None | None |
| ST5789 | None | 1.0 | None |
| ST2146 | None | None | 2.0 |
| ST213 | None | 1.0 | None |
| ST23 | None | 1.0 | None |
| ST269 | None | 1.0 | None |
| ST334 | None | 1.0 | None |
| ST8445 | None | 1.0 | None |
| ST4 | None | 2.0 | None |
| ST2875 | None | 1.0 | None |
| ST461 | None | 1.0 | None |
| ST53 | None | 1.0 | None |
| ST3493 | 1.0 | None | None |
| ST7962 | None | None | 1.0 |
| ST10 | None | 1.0 | None |Number of strains
D
### Chart
| Category | ND | | |
|---|---|---|---|
| A | 1.0 | 25.0 | 9.0 |
| B | 9.0 | 25.0 | 25.0 |
| C | 4.0 | 7.0 | 38.0 |
| cnl | None | 1.0 | None |
| E | None | None | 2.0 |
| I | None | 1.0 | None |
| NG | None | None | 4.0 |
| W | None | 8.0 | 2.0 |
| X | None | 2.0 | 1.0 |
| Y | None | 1.0 | 1.0 |Number of strains
